# Supplementary figures and images for: Autistic traits and alcohol consumption through adolescence and young adulthood
Source: medRxiv. 2025 Nov 25:2025.11.24.25340869. Preprint. [Version 1] doi: 10.1101/2025.11.24.25340869 (PMC12676549; doi:10.1101/2025.11.24.25340869)

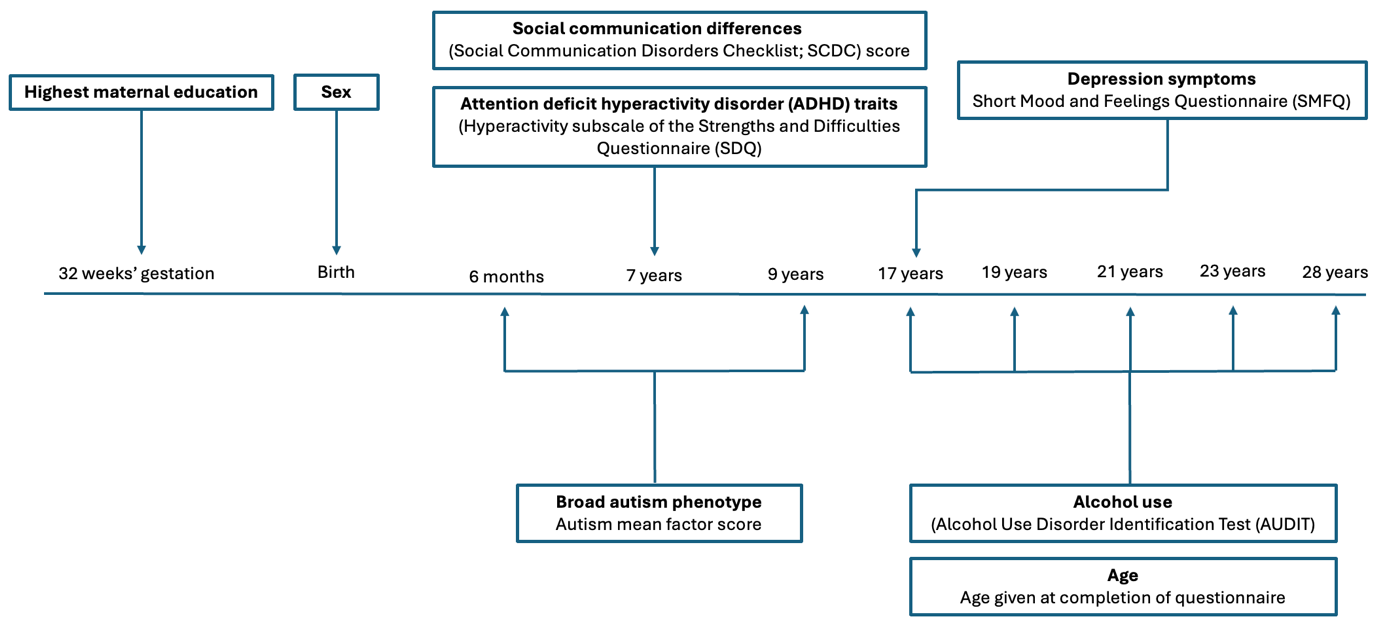

Supplement: Supplement 1 [file media-1.docx]

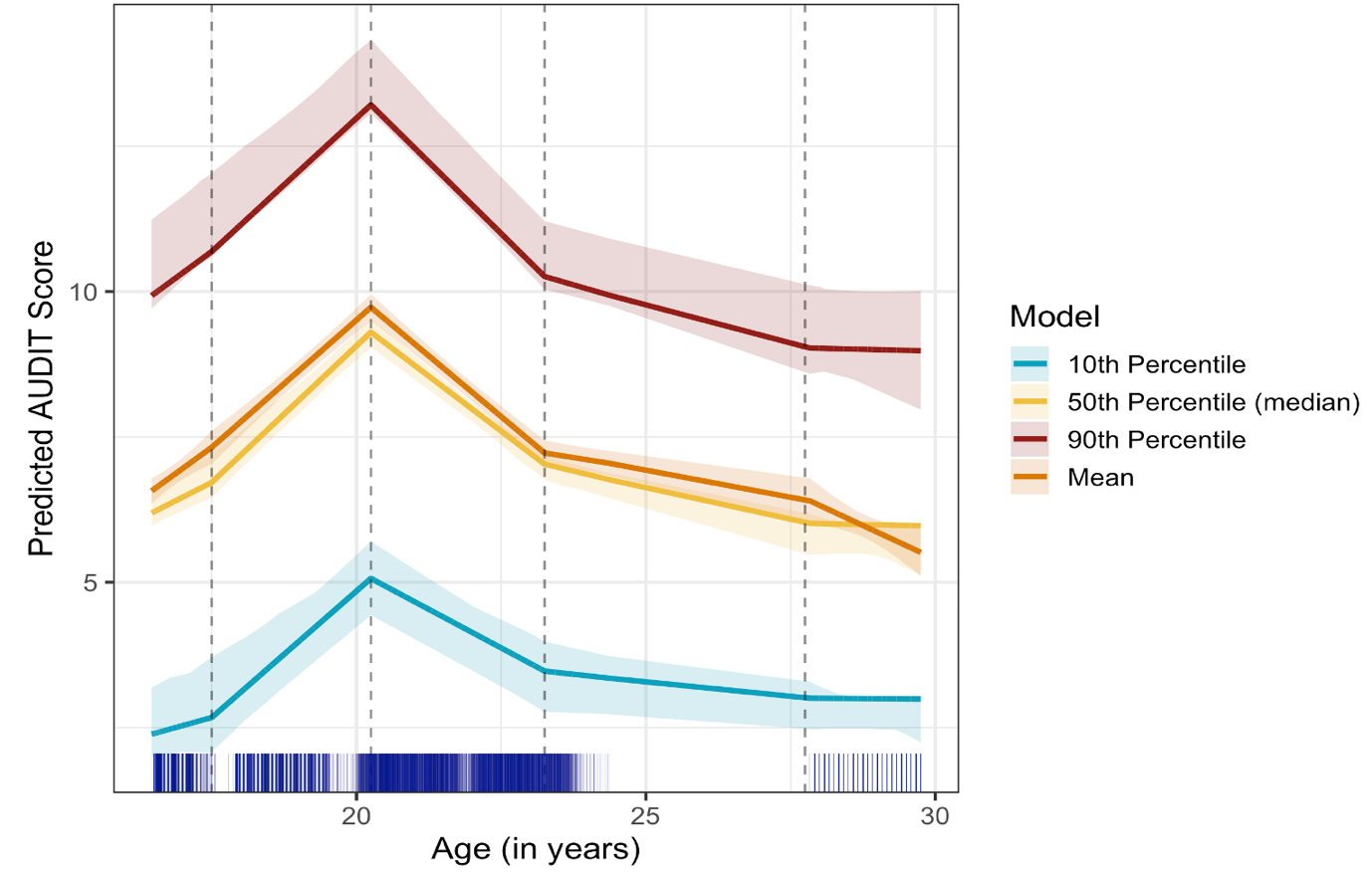

Supplement: Supplement 2 [file media-2.docx]
